# Supplementary material for: Causal Relationship between Aging and Anorexia Nervosa: A White-Matter-Microstructure-Mediated Mendelian Randomization Analysis
Source: Biomedicines. 2024 Aug 16;12(8):1874. doi: 10.3390/biomedicines12081874 (PMC11351342; doi:10.3390/biomedicines12081874)
Supplement: Supplementary file 1 [file biomedicines-12-01874-s001.zip › Supplementary Figure/Figure S4/Singe SNP plot of MR analysis for TL to ubm-b-1850.pdf]

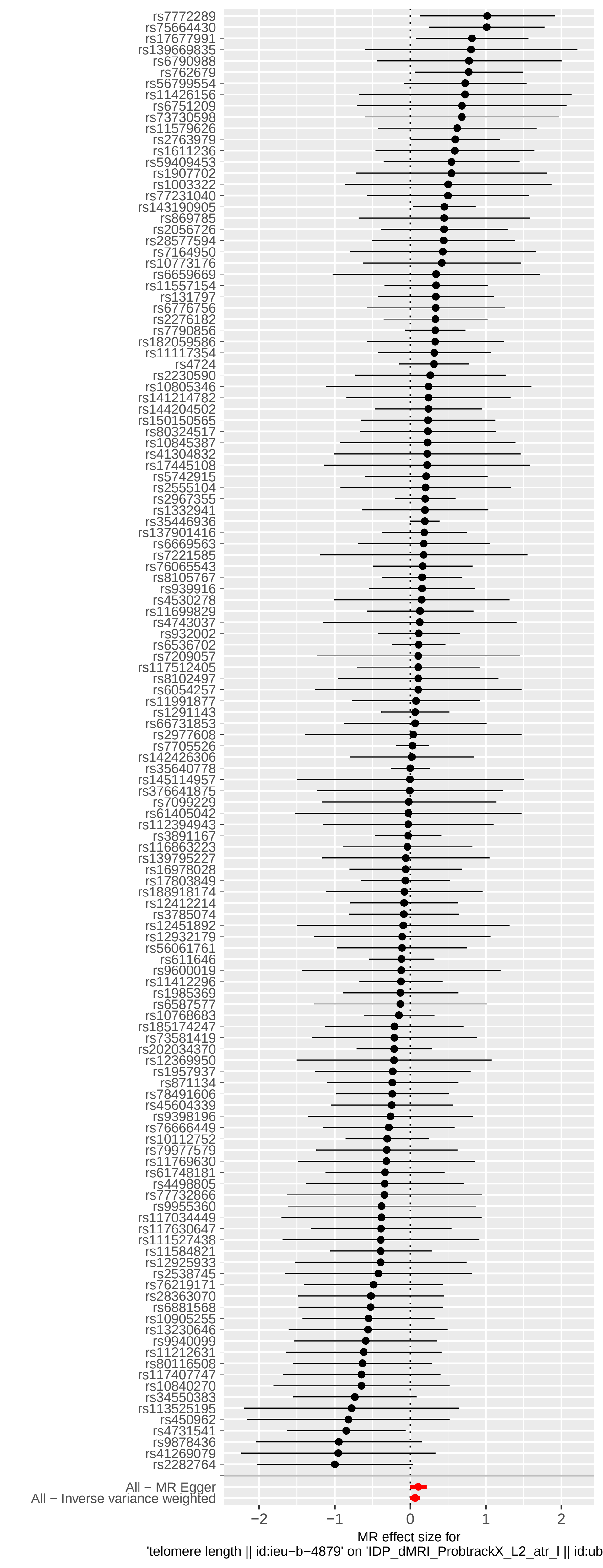
rs7772289
rs75664430
rs17677991
rs139669835
rs6790988
rs762679
rs56799554
rs11426156
rs6751209
rs73730598
rs11579626
rs2763979
rs1611236
rs59409453
rs1907702
rs1003322
rs77231040
rs143190905
rs869785
rs2056726
rs28577594
rs7164950
rs10773176
rs6659669
rs11557154
rs131797
rs6776756
rs2276182
rs7790856
rs182059586
rs11117354
rs4724
rs2230590
rs10805346
rs141214782
rs144204502
rs150150565
rs80324517
rs10845387
rs41304832
rs17445108
rs5742915
rs2555104
rs2967355
rs1332941
rs35446936
rs137901416
rs6669563
rs7221585
rs76065543
rs8105767
rs939916
rs4530278
rs11699829
rs4743037
rs932002
rs6536702
rs7209057
rs117512405
rs8102497
rs6054257
rs11991877
rs1291143
rs66731853
rs2977608
rs7705526
rs142426306
rs35640778
rs145114957
rs376641875
rs7099229
rs61405042
rs112394943
rs3891167
rs116863223
rs139795227
rs16978028
rs17803849
rs188918174
rs12412214
rs3785074
rs12451892
rs12932179
rs56061761
rs611646
rs9600019
rs11412296
rs1985369
rs6587577
rs10768683
rs185174247
rs73581419
rs202034370
rs12369950
rs1957937
rs871134
rs78491606
rs45604339
rs9398196
rs76666449
rs10112752
rs79977579
rs11769630
rs61748181
rs4498805
rs77732866
rs9955360
rs117034449
rs117630647
rs111527438
rs11584821
rs12925933
rs2538745
rs76219171
rs28363070
rs6881568
rs10905255
rs13230646
rs9940099
rs11212631
rs80116508
rs117407747
rs10840270
rs34550383
rs113525195
rs450962
rs4731541
rs9878436
rs41269079
rs2282764
All − MR Egger
All − Inverse variance weighted
−2
−1
0
1
2
MR effect size for
'telomere length || id:ieu−b−4879' on 'IDP_dMRI_ProbtrackX_L2_atr_l || id:ub
